# Taurolidine Lock Is Superior to Heparin Lock in the Prevention of Catheter Related Bloodstream Infections and Occlusions

**DOI:** 10.1371/journal.pone.0111216

**Published:** 2014-11-07

**Authors:** Evelyn D. Olthof, Michelle W. Versleijen, Getty Huisman–de Waal, Ton Feuth, Wietske Kievit, Geert J. A. Wanten

**Affiliations:** 1 Intestinal Failure Unit, Department of Gastroenterology and Hepatology, Radboud University Medical Center, Nijmegen, The Netherlands; 2 Department of Nuclear Medicine, Netherlands Cancer Institute – Antoni van Leeuwenhoek, Amsterdam, The Netherlands; 3 Scientific Institute for Quality of Healthcare, Radboud University Medical Center, Nijmegen, The Netherlands; 4 Department of Health Evidence, Radboud University Medical Center, Nijmegen, The Netherlands; Medical University of Graz, Austria

## Abstract

**Background and Aims:**

Patients on home parenteral nutrition (HPN) are at risk for catheter-related complications; mainly infections and occlusions. We have previously shown in HPN patients presenting with catheter sepsis that catheter locking with taurolidine dramatically reduced re-infections when compared with heparin. Our HPN population therefore switched from heparin to taurolidine in 2008. The aim of the present study was to compare long-term effects of this catheter lock strategy on the occurrence of catheter-related bloodstream infections and occlusions in HPN patients.

**Methods:**

Data of catheter-related complications were retrospectively collected from 212 patients who received HPN between January 2000 and November 2011, comprising 545 and 200 catheters during catheter lock therapy with heparin and taurolidine, respectively. We evaluated catheter-related bloodstream infection and occlusion incidence rates using Poisson-normal regression analysis. Incidence rate ratios were calculated by dividing incidence rates of heparin by those of taurolidine, adjusting for underlying disease, use of anticoagulants or immune suppressives, frequency of HPN/fluid administration, composition of infusion fluids, and duration of HPN/fluid use before catheter creation.

**Results:**

Bloodstream infection incidence rates were 1.1/year for heparin and 0.2/year for taurolidine locked catheters. Occlusion incidence rates were 0.2/year for heparin and 0.1/year for taurolidine locked catheters. Adjusted incidence ratios of heparin compared to taurolidine were 5.9 (95% confidence interval, 3.9–8.7) for bloodstream infections and 1.9 (95% confidence interval, 1.1–3.1) for occlusions.

**Conclusions:**

Given that no other procedural changes than the catheter lock strategy were implemented during the observation period, these data strongly suggest that taurolidine decreases catheter-related bloodstream infections and occlusions in HPN patients compared with heparin.

## Introduction

Catheter-related bloodstream infections (CRBSIs) remain the major, potentially life-threatening, complication of home parenteral nutrition (HPN) therapy. As such, CRBSIs pose a massive burden on the patients'quality of life and hospital resources due to the frequent need for hospital admission, surgical and medical interventions and, eventually, the need for intestinal transplantation when venous access becomes irreversibly compromised [1].

Patient-, therapy- and device-related risk factors for CRBSIs have been characterized previously in detail [2]. The nature of the underlying disease leading to intestinal failure may increase the risk of CRBSI [3]. Also factors that are related to the composition of the parenteral nutrition formulation, such as caloric content and the presence of a lipid emulsion play a role [4], as well as the frequency and duration of the use of the venous access device [5]. The presence of a venous access device that bypasses the natural host barriers by directly connecting the external environment to the patients' central bloodstream, has been identified as an independent risk factor for the occurrence of CRBSIs [6]. The magnitude of the risk also depends on catheter material [5], site of catheter insertion [7], and catheter coating [8]. Finally, the agent that is used to lock the central venous catheter (CVC) after infusion of the parenteral nutrition is increasingly being recognized as pivotal in the prevention of CRBSIs [9].

Several lock solutions, some of which include (combinations of) anticoagulants, fibrinolytic agents, antiseptics and antibiotics, have been introduced, but failed because of side effects, microbial resistance issues or lack in effectiveness [10]. Taurolidine, a microbiocidal agent, has a broad spectrum activity against bacteria and fungi [11]. The suggested microbiocidal activity of taurolidine involves a chemical interaction with the microbial cell wall resulting in irreparable injury [12]. Taurolidine has shown to reduce the risk for CRBSIs in several patient groups who depend on a reliable central venous access device [9], [13]–[19]. A recent meta-analysis confirms these beneficial effects, but also emphasizes low power and methodological flaws of the currently available studies [20].

A randomized trial in our own tertiary HPN referral center comparing the catheter lock strategy using 2% taurolidine (Taurosept) and low dose (150 U/mL) heparin on the recurrence of CRBSIs was preliminary terminated due to the dramatic decrease in CRBSIs in taurolidine locked catheters that became apparent because of the open label character of this study [9]. Although the sample size of this formal randomized trial was very low (heparin: n = 14), taurolidine (n = 16), we considered the results evident enough to switch from low dose heparin to 2% taurolidine catheter locking in all of our HPN patients in the fall of 2008. In the present study, we provide further evidence that taurolidine may be more effective in preventing catheter-related complications in HPN patients compared to heparin, using a comprehensive dataset of long-term catheter locking, covering the period from 2000 to late 2008 (using low-dose heparin) and the period from 2008 to 2011 (using taurolidine). The study comprises 212 patients, 745 central venous catheters and more than 200,000 catheter days, and provides by far the most robust dataset in this field so far. Importantly, none of our HPN practice procedures or materials, other than the catheter lock strategy, changed during the complete observation period.

## Patients and Methods

### Ethics

The research ethics committee of the Radboud University Nijmegen Medical Centre (CMO Regio Arnhem-Nijmegen) approved this retrospective study under the protocol number (CMO: 2014/167). This ethics committee confirmed that individual written informed consent was not needed. Data was collected, anonymized and de-identified by the treating physician (GW). Subsequently data were entered in a database and statistical analysis was performed.

### Patients

We enrolled all consecutive patients on long-term (>3 months and >1/week used for HPN/fluid administration) HPN or fluids using a central venous catheter (Hickman or Port-A-Cath (PAC, implantable port)) at the Radboud University Medical Centre, Nijmegen, the Netherlands, between January 1^st^, 2000 and November 1^st^, 2011 (n = 212). Medical records of all patients were reviewed. Patients were categorized into one of five underlying disease groups, based on the indication for fluid/HPN treatment: motility disorder, short bowel with stoma, short bowel without stoma, impaired intestinal absorption (mainly due to radiation enteritis), or other. Data on time and site of catheter insertion were checked in operation reports.

### Cleaning protocol of catheter

Only single lumen catheters were placed. During the observation period of this study, none of our cleaning protocol procedures changed. In general, patients are trained in procedures including catheter handling and HPN/fluid administration during an inpatient training period of 1 to 2 weeks in our hospital.

Independent of the access type, the patient's or care-givers's hands are washed and subsequently disinfected with chlorhexidine in ethanol prior to HPN/fluid administration or cleaning of the exit site or skin. Cleaning protocols for Hickman catheter and PAC in our hospital are described in more detail in the following text.

For Hickman catheters, the catheter exit site is covered direct after catheter insertion with a Tegaderm pad (3M Health Care, Neuss, Germany) which is replaced every 96 hours. The exit site is disinfected by circular movements from the exit site to the outer circumference. Every circle is disinfected with a new sterile swap. The first 10 centimeters of the catheter is disinfected with a sterile swap and chlorhexidine in ethanol. After drying, the exit site is covered with a new Tegaderm pad. After 3 weeks, the catheter cuff has grown in the surrounding tissues and sutures for line fixation are removed. From then on, the exit site is no longer covered with a Tegaderm pad and is cleaned daily by washing with water and soap and drying with a clean towel.

For PACs, after placement the wound is covered with sterile compresses. Before HPN/fluid administration, the skin overlying the subcutaneous port is cleaned with chlorhexidine by the described circular movement method above. After HPN administration, the puncture-site is covered with a sterile compress for a few minutes, until (minor) bleeding ends.

### Catheter lock solution

Central venous catheters were locked after every administration of HPN or fluids. After a successful open label randomized controlled trial [9], all HPN patients with a Hickman or PAC switched from low dose (150 U/mL) heparin to 2% taurolidine (Taurosept) in 2008. Of every individual patient the date of switching from heparin to taurolidine was used for data analysis.

**Figure 1:**
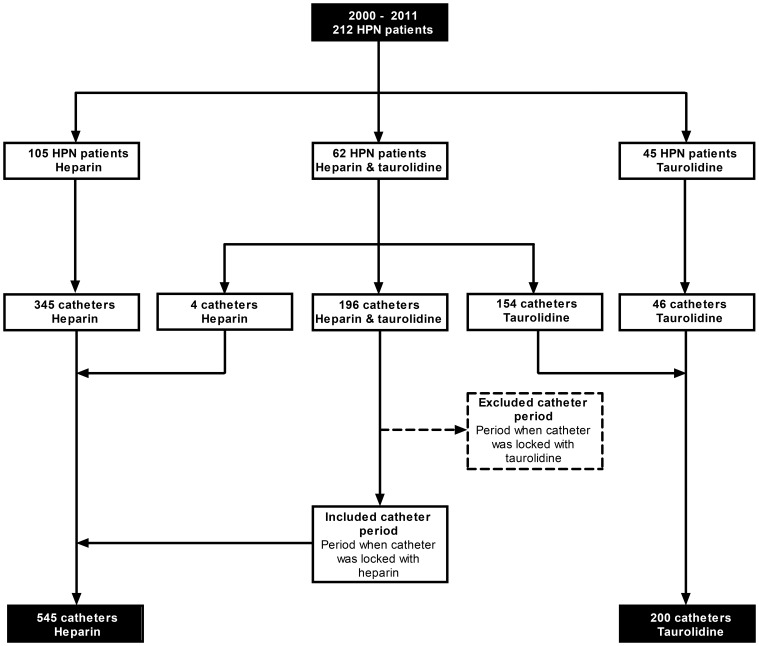
Flowchart of numbers of patients and catheters.

In case a catheter was locked with heparin and subsequently with taurolidine, only the period before starting taurolidine was analyzed, since catheter related complications after start of taurolidine could possibly be a consequence of a carry-over effect of locking with heparin, since a biofilm that originated from the heparin period might lead to a CRBSI in the taurolidine period. In that case, the total number of catheter days was calculated from insertion of the catheter to the starting date of the catheter lock solution taurolidine (Figure 1).

### Catheter related bloodstream infections and occlusions

CRBSIs were defined by the presence of symptoms (fever, chills) associated with positive blood cultures (blood drawn from peripheral vein and/or from venous access device) in the absence of other evident infectious foci that likely could explain the bloodstream infection. Episodes with fever and/or chills without positive blood cultures were only considered a bloodstream infection in case blood samples were drawn under antibiotic treatment (possibly leading to negative blood cultures) and in case patients were showing signs of sepsis [5]. The date of the CRBSI was the date of the first positive blood culture. A CRBSI was considered polymicrobial when different pathogens were found in the peripheral and/or venous access blood culture. All pathogens, were classified as Gram positive or Gram negative bacteria or yeast.

Catheter occlusions were defined by an obstruction of the central venous catheter, described in the medical record, or a vascular occlusion of more than 50% of a vein near the insertion side of the central venous catheter, described in report of a duplex scan. The date of occlusion was the date of the first time that this occlusion was described.

### Treatment

Data on the use of anticoagulants and immune suppressive medication, and the type of HPN/fluid formulation and the frequency of HPN/fluid administrations per week was obtained from pharmacy prescriptions. Catheters were described as catheters which were exposed to only fluids, only HPN or a combination of both.

### Hospital admission

We evaluated hospital admission data in the pre (2006–2007) and post (2009–2011) taurolidine eras. The transition year 2008 included hospital admissions with taurolidine and heparin locked catheters. No detailed hospital admission data were available of the period before 2006. All hospital admissions for in-hospital training of aseptic catheter techniques were excluded from analysis, since these admissions were not due to complications. Hospital admission were presented as the days that the patients were admitted to our ward in one year divided by the total number of catheter days in that year.

### Adverse events

Adverse events resulting in discontinuation of the use of taurolidine were reported. If possible, patients switched first to another taurolidine-containing lock in the form of taurolidine-citrate (Taurolock) and in case of repeated adverse effects switched to saline.

### Statistical analysis

Characteristics of patients, vascular accesses and CRBSIs were presented as number (n) with percentage (%), or mean with 95% confidence interval (CI), or median with 25^th^ and 75^th^ percentile. Chi-square test for categorical data and Mann Whitney U test for continuous data were used to compare the heparin and taurolidine group in Table 1 and 2. Statistical significance was accepted if the probability of a type I error did not exceed 5%. We analyzed incidence rates and incidence rate ratios (i.e., the complication incidence rate that occurred with catheters locked with heparin divided by those locked with taurolidine) of CRBSIs and occlusions using random effects model with Poisson distributions for counts. Random effects for patients were incorporated in the modeling process to account for repeated vascular access periods within a patient. Possible confounders in the relation between catheter lock solution and complication rate were identified and reported, based on biological and clinical rationales [5], or a change of 10% or more on unadjusted complication rate ratios by a covariate. Adjusted complication incidence rate ratios for heparin over taurolidine were calculated with respect to these confounders. The NLMIXED procedure of SAS System for Windows version 9.2 was used (SAS Institute Inc, Cary, NC). Statistical significance was accepted if the probability of a type I error did not exceed 5%. To provide information about statistical significance, we reported 95% CI where appropriate. In case data were missing from medical records these were considered to be completely at random and were excluded from analyses.

**Table 1:** Vascular access characteristics.

	Heparin (n = 545)	Taurolidine (n = 200)	p-value
**Duration of HPN/fluid use before creation catheter (days: median (25^th^–75^th^ percentile))**	214 (34–765)	564 (103–1489)	0.000*
Unknown (n (%))	7 (1)	0 (0)	
**Type of vascular access (n (%))**			0.536
Hickman	368 (68)	140 (70)	
Port à cath	177 (32)	60 (30)	
**Place of catheter insertion (n (%))**			0.000*
Subclavian vein	337 (62)	57 (29)	
Jugular vein	63 (12)	105 (52)	
Femoral vein	33 (6)	19 (9)	
Inferior caval vein	13 (2)	6 (3)	
Unknown	99 (18)	13 (7)	
**Catheter survival (days)**			0.000*
Median (25^th^–75^th^ percentile)	120 (43–310)	209 (65–611)	
Total number of catheter days of all catheters	147,842	71,112	
**Number of catheters still in place (n (%))**			0.000*
Yes	1 (0)	80 (40)	
No	544 (100)	120 (60)	
**Composition of infusional fluid (n (%))**			0.380
HPN alone	290 (53)	118 (59)	
Fluid alone	30 (6)	16 (8)	
HPN & fluid	178 (32)	61 (31)	
Unknown	47 (9)	5 (2)	
**HPN/fluid administration frequency, per week (n (%))**			0.529
1	3 (1)	1 (1)	
2	5 (1)	4 (2)	
3	32 (6)	14 (7)	
4	34 (6)	18 (9)	
5	50 (9)	21 (11)	
6	44 (8)	11 (5)	
7	319 (58)	111 (55)	
Unknown	58 (11)	20 (10)	
**Immune suppressive use (n (%))**			0.121
Yes	83 (15)	41 (21)	
No	437 (80)	154 (77)	
Unknown	25 (5)	5 (2)	
**Anticoagulant use (n (%))**			0.932
Yes	288 (53)	111 (55)	
No	220 (40)	83 (42)	
Unknown	37 (7)	6 (3)	

Variables are shown per vascular access. Patients may have had multiple vascular accesses, total number of patients assessed is 212 (Figure 1). Characteristics were presented as number of events (n) with percentage (%) or median with 25^th^ and 75^th^ percentile. A p-value lower than 0.05 was considered statistically significant.

**Table 2:** Characteristics of catheter related bloodstream infections.

	Heparin	Taurolidine	p-value
**Origin positive blood cultures (n (%))**			0.000*
Peripheral	34 (7)	15 (35)	
Catheter	217 (47)	9 (21)	
Both	206 (44)	19 (44)	
Unknown	6 (1)	0 (0)	
**Type of bloodstream infection (n (%))**			0.147
** **Monomicrobial	345 (74)	28 (65)	
Polymicrobial	113 (24)	15 (35)	
Unknown	6 (2)	0 (0)	
Type of pathogens in peripheral blood culture (n (%))**			0.093
Gram positive bacteria	164 (57)	24 (55)	
Gram negative bacteria	94 (32)	11 (25)	
Yeast	26 (9)	9 (20)	
Unknown	6 (2)	0 (0)	
Type of pathogens in catheter blood culture (n (%))**			0.332
Gram positive bacteria	313 (55)	24 (57)	
Gram negative bacteria	209 (37)	12 (29)	
Yeast	43 (7)	6 (14)	
Unknown	6 (1)	0 (0)	

Characteristics were presented as number of events (n) with percentage (%). *A p-value lower than 0.05 was considered statistically significant. **More pathogens than CRBSI, because of polymicrobial infections and differences in positive blood cultures between blood cultures of peripheral and venous access origin.

## Results

### Study population

212 HPN patients were included in the study: 62 patients had multiple catheters that were initially locked with heparin and later catheters that were locked with taurolidine, 105 patients had catheters that were exclusively locked with heparin, and 45 patients had their catheters exclusively locked with taurolidine (Figure 1). Most patients were male (n (%): 102 (61%) and 74 (69%) in heparin and taurolidine group, respectively). Patients started HPN at a mean (95% CI) age of 48 years (18–78 years) in the heparin group and 49 years (22–77 years) in the taurolidine group. The major indications for HPN (n (%)) use were motility disorder and short bowel syndrome with or without a stoma; 46 (28%), 32 (19%) and 69 (41%) patients in the heparin group, and 40 (37%), 28 (26%) and 34 (32%) patients in the taurolidine group, respectively. Less common indication of HPN was impaired intestinal absorption; 9 (5%) and 4 (4%) patients in the heparin and taurolidine group, respectively. All remaining patients were classified as other HPN indication (n (%)); 11 (7%) patients with heparin locked catheters and 1 (1%) patient with taurolidine locked catheters. The majority of patients (n (%)) was trained at our tertiary referral centre in aseptic catheter handling and parenteral nutrition administration; this concerned 143 (86%) patients for the heparin and 83 (78%) patients for the taurolidine group.

### Vascular accesses

The characteristics of a total of 745 central venous catheters, of which 545 were locked with heparin and 200 with taurolidine were included (Figure 1) and analyzed (Table 1). In both groups Hickman catheters (about 70%) were used more frequently than PACs (about 30%). Around 75% of the catheters were inserted in the jugular or subclavian veins; in the heparin group the subclavian vein (62%) was the most common insertion place, while in the taurolidine group the jugular vein (52%) was mostly used. Catheter survival (days: median (25^th^–75^th^ percentile)) was longer in the taurolidine group (209 days (65–611 days)) compared to the heparin group (120 days (43–310 days)). The total number of catheter days was 147,842 for the 545 heparin locked catheters and 71,112 for the 200 taurolidine locked catheters. Patients with catheters in the taurolidine group were more experienced in the administration of HPN/fluids (probably a consequence of having had heparin locked catheters before), since the median (25^th^–75^th^ percentile) duration from the start of HPN/fluid use to the creation of the venous access was longer; 214 days (34–765 days) for the heparin group and 564 days (103–1489 days) for the taurolidine group. Most catheters were used more than 5 days a week (75% and 71%, in the heparin and taurolidine group, respectively). HPN only was administered in 53% and 59%, in the heparin and taurolidine group, respectively, whilst a combination of HPN and fluids was administered in 32% and 31%, respectively. A minority, 6% of heparin and 8% of taurolidine catheters, was used for fluid administration only. Half of the catheters (53% and 55% in heparin and taurolidine group, respectively) were inserted in patients who used anticoagulants, and less than a quarter (15% and 21% in heparin and taurolidine group, respectively) of the catheters were inserted in patients who used immune suppressive medication.

### Bloodstream infections and occlusion incidence rates

CRBSIs were 464 and 43 times detected in heparin and taurolidine locked catheters, respectively. Table 2 presents the characteristics of these CRBSIs. Forty-four percent of CRBSIs had both a positive blood culture of peripheral and venous access origin. The majority of CRBSIs (74 and 65% in heparin and taurolidine group, respectively) was based on a single type of pathogen. The most common microbial species that caused these CRBSIs were Gram-positive bacteria, followed by Gram-negative bacteria and fungi.

Table 3 shows the unadjusted incidence rates and the adjusted (for confounders) incidence rate ratios of heparin over taurolidine. Bloodstream infection incidence rates (per access year (95% CI)) were 1.1 per access year (0.9–1.3 per access year) in the heparin group and 0.2 per access year (0.1–0.2 per access year) in the taurolidine group. The bloodstream infection incidence ratio, of heparin compared to taurolidine incidence rates, adjusted for confounders (underlying disease, anticoagulant use, immune suppressive use, HPN/fluid frequency per week, composition of infusional fluid, place of catheter insertion and HPN/fluid use before creation catheter), was 5.9 (95% CI, 3.9–8.7).

**Table 3:** Catheter related bloodstream infection and occlusion incidence rates and incidence rate ratios in heparin and taurolidine locked catheters.

	Incidence rate per access year (95% CI)	Adjusted incidence rate ratio (95% CI)*
**Catheter related bloodstream infection**		
Heparin	1.1 (0.9–1.3)	
Taurolidine	0.2 (0.1–0.2)	
Heparin/taurolidine ratio		**5.9 (3.9–8.7)**
**Catheter related occlusion**		
Heparin	0.2 (0.2–0.3)	
Taurolidine	0.1 (0.1–0.2)	
Heparin/taurolidine ratio		**1.9 (1.1–3.1)**

Data were analyzed using random effects model with Poisson distributions for counts. *Adjusted values are corrected for: underlying disease, anticoagulant use, immune suppressive use, HPN/fluid frequency per week, composition of infusional fluid, and duration of HPN/fluid use before creation catheter. Random effects for patients were incorporated to account for repeated vascular access periods within a patient.

Catheter related vascular occlusions were 137 and 34 times detected in heparin and taurolidine locked central venous catheters, respectively. As presented in Table 3, occlusion rates (per access year (95% CI)) were slightly lower in the taurolidine group (0.1 per access year (0.1–0.2 per access year)) compared to the heparin group (0.2 per access year (0.2–0.3 per access year)). The occlusion incidence ratio, of heparin compared to taurolidine incidence rates, adjusted for confounders (underlying disease, anticoagulant use, immune suppressive use, HPN/fluid frequency per week, composition of infusional fluid, place of catheter insertion and HPN/fluid use before creation catheter), was 1.9 (95% CI, 1.1–3.1).

### Hospital admissions

**Figure 2:**
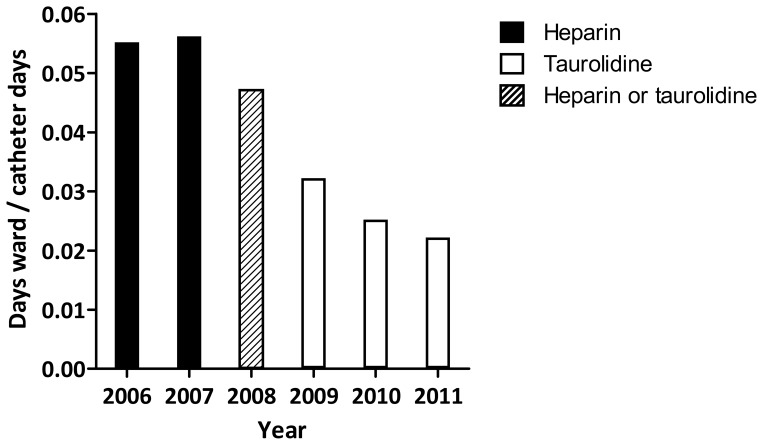
Hospital admissions in period 2006 until 2011. Catheters were locked with heparin (2006–2007) and taurolidine (2009–2011). In 2008 admissions of both lock strategies were included. Data are presented as the days that the patients were admitted to our ward divided by the total number of catheter days.

From 2006 towards the end of 2011 the number of HPN-related admission days at our 15-bed clinical ward remained stable at a mean (± SD) of 1173±159 days per year, while the HPN population increased from 61 to 133 patients, hence the number of catheter days increased from 21,619 to 45,960. Therefore, the ratio of hospital admission days per catheter day decreased by 60% from 0.055 in two pre-taurolidine years to 0.022 in 2011 (Figure 2).

### Adverse events

Since the switch from heparin to taurolidine 8 of the 107 patients reported adverse events possibly related to use of taurolidine, and did not continue using pure taurolidine as a result. One patient experienced an anaphylactic-like reaction (vomiting, shortness of breath and urticaria) within minutes after the first administration and switched to saline for catheter locking. Five patients, who experienced a burning sensation, PAC occlusion, dizziness, nausea or pain and paresthesia, switched first to taurolidine-citrate, but experienced similar adverse reactions and switched thereafter successfully to saline. Two patients who experienced palpitations or discomfort over the chest switched successfully to taurolidine-citrate.

## Discussion

The availability and maintenance of an adequate and reliable venous access remains the foremost worry in long-term HPN care, with catheter related complications, mainly infections and occlusions, causing the majority of problems. This study presents by far the most robust data set so far to demonstrate that the use of taurolidine as catheter lock versus low-dose heparin decreases catheter related complications. During an observation period spanning over 200,000 catheter days, we found an impressive six times higher chance of developing CRBSI in heparin compared to taurolidine locked catheters. Also, a two times higher risk for developing catheter occlusions in heparin locked compared to taurolidine locked catheters was observed. Interestingly, we saw that this decrease in catheter complications was accompanied by a steep decrease in strain on healthcare resources in the form of diminished hospital admissions. Importantly, none of our other HPN-related procedures or techniques changed during this period suggesting that the type of catheter lock was instrumental in these observations.

The observed effectiveness of taurolidine to prevent CRBSI found in our study is in agreement with previous research in various patient populations [9], [21]–[25]. A meta-analysis including six studies covering 86,000 catheter days found a slightly lower than our study, but still an impressive three times (confidence interval: 1.8–4.8) higher risk for the development of CRBSI in CVCs locked with heparin compared to taurolidine. Due to small sample sizes as well as methodological deficiencies of the included studies in the meta-analysis these results should be interpreted with caution [20]. Also because these studies contain different heterogeneous patient populations with distinct risk profiles for the development of CRBSIs, and because the effects of diverse taurolidine containing lock solutions, which differ in taurolidine concentration and the presence of other agents such as citrate and/or heparin, were pooled. Concerning the latter, minor differences between extremely diluted pure taurolidine and taurolidine-citrate(-heparin) solutions in the inhibition of growth of yeast, Gram negative and Gram positive bacteria *in vitro* have been found. The clinical relevance of these minor differences between different taurolidine solutions remains however unclear in the absence of clinical comparative studies [26].

Although taurolidine has shown to be able to decrease thrombus weight, but is not as effective as heparin in this respect [27], we observed a lowered incidence of catheter related occlusions with its use. Previous studies described a relationship between the number of CRBSI and occlusions, possibly due to infection-induced activation of the coagulation system [28]. In the same vein, the decrease in catheter related occlusions in our study may be the result of diminished vascular damage because of the lower infection rate [28]. In contrast, the earlier mentioned meta-analysis (with its described limitations) found no significant difference between taurolidine and heparin-treated CVCs in incidence of catheter occlusions [20].

The sharp decrease in catheter related complications that we observed after switching from catheter locking with heparin to taurolidine had a highly significant impact on the clinical burden that HPN care imposes on our clinical ward, as proven by the sharp decrease in number of days that HPN patients spent within the hospital. Keeping in mind that in Europe in general the cost of each case of catheter infection lies between 4,000 to 13,000 Euros [29], and the costs in the Netherlands for one year taurolidine locking (1,800 Euro per year) are about 300 Euros more than for heparin locking (1,500 Euro per year), taurolidine seems from a financial point of view promising. Still, a formal cost-effectiveness analysis is necessary to confirm that taurolidine is cost-effective.

In our study, approximately 7 percent of all patients who locked their catheter with taurolidine experienced (mostly mild) side effects, that urged us to stop the use of (any) taurolidine or switch to a different taurolidine formulation. This switch mostly resulted in similar side effects after which the patient used saline as a catheter lock. Only one probable anaphylactic reaction was observed and we did not dare to rechallenge this patient with the same or another taurolidine preparation. Although theoretically anaphylaxis seems unlikely due to the metabolization of taurolidine into taurine and carbon dioxide, other constituents such as polyvinylpyrrolidone (PVP-17), that is used as a stabilisator/emulgator, might cause these problems, as described in a case study [30]. Side effects have been described before for taurolidine with citrate in a paediatric patient population with haematological malignancies. Twenty percent of the paediatric patients had side effects ranging from discomfort in chest and neck, perioral dysaesthesia, abnormal taste sensations to nausea, and half of these patients using the lock solution taurolidine with citrate was urged to stop with these lock solution because of the side effects they experienced [22].

The statistical significant differences between the taurolidine and heparin locked catheters in HPN/fluid use before the creation of the catheter and the vascular acces *in situ* duration (Table 1) are a direct consequence of the fact that 62 of the 212 patients first locked their catheters with heparin and subsequently locked with taurolidine (Figure 1). The number of catheters that are still *in situ* are significantly higher in the taurolidine group (Table 1), primarily because the HPN population switched to taurolidine and none of the catheters were locked with heparin at the end of this study.

The place of catheter insertion is significantly different between the two groups. However, the place of insertion is dependent of different factors. The choice of venous access depends on the estimated length of parenteral nutrition dependency, the condition of the veins and the personal preference of the patient, with respect to esthetics (visibility of access device) and need for (self-)puncturing. Patients who have had more complications, may have less options left for venous access. A comparison between the place of insertion between catheters locked with taurolidine and heparin is therefore difficult. A recent systematic review did find similar risks for catheter-related complications in subclavian and internal jugular central venous catheters [31].

A significant difference was found in the origin of positive blood cultures between heparin and taurolidine locked catheters (Table 2). We could not explain these differences. When we compare the type of pathogens that caused the CRBSI in the heparin and the taurolidine locked catheters (Table 2), it is interestingly to observe that in taurolidine locked catheters a higher percentage of the CRBSIs is caused by yeasts. This might theoretically be a consequence of the fact that yeast are less sensitive to taurolidine, since *in vitro* studies showed that higher taurolidine concentrations are necessary to eliminate yeasts compared to bacteria [11].

The results of the statistical analysis in Table 1 and 2 should be interpreted with care, since the variables were not corrected for overrepresentation of patients who had multiple vascular accesses. To control for overrepresentation of certain patients, we used a specific statistical model that accounts for repeated measurements in the Poisson analysis, presented in Table 3. Variables of Table 1 and 2 were added as a covariate in the Poisson analysis, based on biological and clinical rationales, or based on the fact that the covariate changed the unadjusted complication rate ratios with 10% or more.

The retrospective nature of this study obviously has its pro's and con's. This study setting enabled us to collect and analyze a substantial number of patients (212), catheters (745) and catheter days (>200,000) from a single center, which makes this by far the most extensive study in this field so far, with only a limited amount of missing data. The downside remains that this study setting hampers the drawing of any conclusions on causal relations.

A limitation of the study is that the diagnoses was based on the presence of symptoms (fever, chills) in association with positive blood cultures and in the absence of other evident infectious foci that likely could explain such an infection. Because of the retrospective nature of the study, it was not possible to use the differential-time-to positivity criteria for diagnoses of CRBSI.

We chose to include all venous accesses of a single patient instead of only the first, since most long-term HPN patients are likely to have more than one catheter over, making our approach more representative for clinical practice. Since none of our other HPN-related procedures or techniques changed during this period, except for the locking strategy, we don't think that bias was introduced by the fact that the cohorts of heparin and taurolidine catheters had not the same observation period.

An important remaining question is whether taurolidine should be used in all HPN patients or only in those who have a high risk for developing CRBSIs, also in light of the fact that it remains unclear from our study design whether the difference in infection rates between both strategies is caused by promotion of infections by heparin and/or prevention of this problem by taurolidine. We hope to shed light on this issue in a multicenter randomized controlled trial that is currently ongoing and which investigates the effectiveness of taurolidine versus saline in preventing CRBSI (ClinicalTrials.gov number, NCT01826526).

Despite the limitations of the study, we suggest that the long-term use of the lock solution taurolidine is more effective in preventing catheter related bloodstream infections and occlusions in HPN patients with CVCs than heparin.

## Supporting Information

File S1 Raw data Incidence Rate Ratio.
(SAV)

File S2 Raw data CRBSI.
(SAV)

File S3 Raw data Hospital Admission.
(XLSX)
